# Characterisation of a Novel Acetyl Xylan Esterase (BaAXE) Screened from the Gut Microbiota of the Common Black Slug (*Arion ater*)

**DOI:** 10.3390/molecules27092999

**Published:** 2022-05-07

**Authors:** Henry Madubuike, Natalie Ferry

**Affiliations:** School of Science, Engineering and Environment, University of Salford, Manchester M5 4WT, UK

**Keywords:** acetyl xylan esterase, lignocellulose, hemicellulose, novel enzyme bioprospecting, carboxylesterase, hydrolase

## Abstract

Acetyl xylan esterases (AXEs) are enzymes capable of hydrolysing the acetyl bonds in acetylated xylan, allowing for enhanced activity of backbone-depolymerizing enzymes. Bioprospecting novel AXE is essential in designing enzyme cocktails with desired characteristics targeting the complete breakdown of lignocellulose. In this article, we report the characterisation of a novel AXE identified as Gene_id_40363 in the metagenomic library analysed from the gut microbiota of the common black slug. The conserved domain description was identified with an NCBI BLASTp search using the translated nucleotide sequence as a query. The activity of the recombinant enzyme was tested on various synthetic substrates and acetylated substrates. The protein sequence matched the conserved domain described as putative hydrolase and aligned closely to an uncharacterized esterase from *Buttiauxella agrestis*, hence the designation as BaAXE. BaAXE showed low sequence similarity among characterized CE family proteins with an available 3D structure. BaAXE was active on 4-nitrophenyl acetate, reporting a specific activity of 78.12 U/mg and a Km value of 0.43 mM. The enzyme showed optimal activity at 40 °C and pH 8 and showed high thermal stability, retaining over 40% activity after 2 h of incubation from 40 °C to 100 °C. BaAXE hydrolysed acetyl bonds, releasing acetic acid from acetylated xylan and β-D-glucose pentaacetate. BaAXE has great potential for biotechnological applications harnessing its unique characteristics. In addition, this proves the possibility of bioprospecting novel enzymes from understudied environments.

## 1. Introduction

The utilisation of fossil fuels has led to several concerns, such as greenhouse gas emissions, environmental pollution, and resource depletion, prompting the development of renewable energy alternatives, such as lignocellulose biomass, to mitigate the disadvantages associated with fossil fuel use [1,2,3]. Lignocellulose biomass (LCB) is the most abundant and widely distributed organic chemical on earth [4]. LCB includes hardwood, such as poplar and oak; softwood, such as pine and spruce; agricultural wastes, such as wheat straw and sugarcane bagasse; and dedicated energy crops, such as jatropha and *Miscanthus* sp. [5,6]. LCB is a potential and promising feedstock source for the production of fuels and platform chemicals in the concept of biorefinery [7]. Refining LCB from various sources would produce cellulose and hemicellulose, which can be processed into fuels and chemicals, and lignin, which can be harnessed to produce energy and chemicals [8]. However, the full utilisation of LCB is challenged by its recalcitrant nature, and the need for expensive pretreatment to release the components of LCB makes the technology unsustainable [9,10]. Pretreatment methods, such as physical and chemical pretreatments, are energy-consuming, require special equipment, and produce compounds that can inhibit hydrolytic enzymes and fermentation [11]. For instance, acid treatment of LCB leads to the formation of inhibitory compounds, such as furfural and 5-hydroxyl furfurals, and a large amount of basic solution is required to restore the pH needed for downstream processes [8]. Biological pretreatment with isolated enzymes or enzyme cocktails offers a sustainable pretreatment alternative characterized by a low energy requirement and selective isolation of the different components of LCB and devoid of the formation of inhibitory compounds. Hence, effective biological pretreatment presents a promising approach to achieving LCB degradation with positive economic and environmental impacts [12].

**Figure 1:**
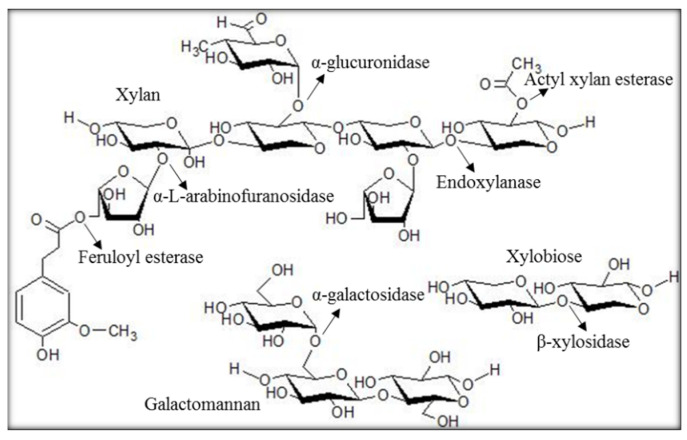
Hemicellulose subunits and breakdown. Structure of major xylan types and enzymes involved in subunit breakdown are identified with arrows indicating enzyme specificity.

Hemicellulose is the second-most abundant polysaccharide type, composed of pentoses, such as xylose, and hexoses, such as glucose [13]. It is an important plant-derived polysaccharide with applications in the development of bio-based chemicals, biofuels, pharmaceuticals, and prebiotics [14,15]. Hemicellulose is heterogeneous and comprises different subunits, such as glucomannans, arabinoglucuronoxylans, xyloglucans, mannans, xylan, and arabinogalactans. The composition of hemicellulose subunits is varied among the various biomass sources [16,17,18]. For instance, hardwoods are essentially composed of xylans in the form of acetylated xylan and glucomannans [19]. The enzymatic hydrolysis of hemicellulose involves various hemicellulases, such as xylanases (endo- and exo-xylanases); β-xylosidases; and accessory enzymes, such as arabinofuranosidases and acetyl xylan esterases (Figure 1) [20]. Xylanases act on the xylan chain to release shorter chains, such as xylooligosaccharide and xylobiose, which are then hydrolysed to xylose units by β-xylosidase [21]. The xylan backbones are often substituted with different side chains, such as acetyl, 4-O-methyl glucuronic acid, feruloyl and ρ-coumaroyl, and arabinosyl residues [14,22]. Acetylation, like other substitutions, restricts the access of xylanases, resulting in reduced release of sugars available for fermentation [23]. Hence, the removal of xylan substitutions is crucial for improved hydrolysis of hemicellulose. The treatment of LCB with accessory enzymes has been shown to increase hydrolysis efficiency and improve the activity of backbone depolymerizing enzymes [23]. For example, the synergetic action of acetyl xylan esterase from *Neocallimastix patriciarum* and xylanase (XynA) released higher amounts of reducing sugars compared with hydrolysis with xylanase alone [24].

Acetyl xylan esterases (AXEs; EC 3.1.1.72) are accessory enzymes able to hydrolyse ester linkages, liberating acetic acid from acetylated hemicellulose [25]. Hardwoods are highly acetylated, with approximately 50–70% of their xylose units acetylated at the C-2 and/or C-3 hydroxyl positions [26]. Hence, acetyl residues are considered the most abundant substitution in hemicellulose [27]. AXEs belong to the α/β hydrolase superfamily, characterised by the α/β- hydrolase fold and the canonical catalytic triad Ser-His-Asp [28]. The sequence of AXEs has the consensus motif Gly-X-Ser-X-Gly around the active site serine [28]. According to the CAZy classification, AXEs are classified into nine carbohydrate esterase (CE) families—CE 1–7, 12, and 16, with the CE 10 family reported as esterases acting on non-carbohydrate substrates [28]. Substrate characterisation of AXEs shows varied substrate activity. Most of the characterised AXEs hydrolysed acetylated xylooligosaccharides and were active on acetylated oligosaccharides/monosaccharides, such as acetylated glucose [29]. For instance, a novel CE (BD-FAE) showed bifunctional attributes, with acetyl xylan esterase activity on acetylated glucuronoxylan from birchwood and feruloyl esterase activity on feruloylated xylooligosaccharides from corn fibre [16]. AXEs have been discovered from a wide range of microbial sources (fungi and bacteria), such as *Thermotoga maritima*, *Bacillus pumilus*, *Trichoderma reesei*, *Clostridium thermocellum*, *Coriolus versicolor*, *Schizophyllum commune*, *Aspergillus versicolor*, *Streptomyces albicans* [12,30], and plant and animal sources [31]. 

Novel AXEs have been mined from different environmental sources such as compost, termite gut, and bovine rumen returning libraries of putative enzymes using a metagenomic approach [32,33]. Although several AXEs have been identified and characterised, the importance of AXEs in lignocellulose degradation supports the need for continuous bioprospecting of novel AXEs with improved properties. The identification of novel AXEs with improved catalytic properties promotes a robust enzyme reservoir for plant biomass degradation under operating conditions [34]. For example, a novel acetyl esterase targeting 4-O-methylglucopyranosyluronic acid substitution in glucuronoxylan was identified from a polysaccharide utilization locus (PUL), having been identified as a protein of unknown function [35]. The common black slug (*Arion ater*) is a plant-feeding organism and one of the most widespread plant pest species in Western Europe and North America [36]. The gut microbiota metagenomics of this organism showed a community of bacteria species known for lignocellulose degradation and well-characterised bacteria plant pathogens [37]. Over 3383 genes with putative activity in lignocellulose degradation were identified from the functional metagenomics screening of this organism [37]. In this work, we report the biochemical and functional properties of a novel AXE identified from the metagenomic library of the common black slug. 

## 2. Results

### 2.1. Bioinformatics and Homology Modelling

**Figure 2:**
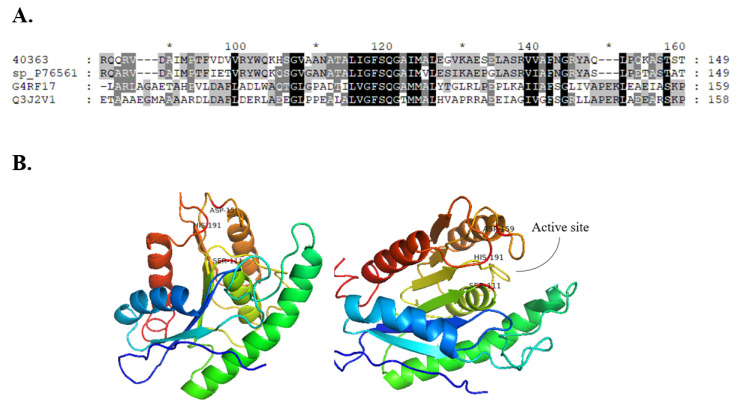
MSA and homology model. (**A**) The sequence of Gene_id_40363 was aligned with selected members of the abhydrolase_2 protein family. The function and source of aligned sequences (from top to bottom) are 40363 (Gene_id_40363, metagenomic sequence), sp_P76561 (esterase YpfH from *Escherichia coli*), Q3J2V1 (phospholipase/carboxylesterase from *Rhodobacter sphaeroides*), and G4RF17 (phospholipase/carboxylesterase from *Pelagibacterium halotolerans*). (**B**) The homology model of Gene_id_40363 was developed in I-Tasser and displayed in PyMOL. Catalytic residues are annotated as S111, D159, and H191.

Gene_id_40363 was annotated as a Carbohydrate Esterase 1 (CE1) family protein in the metagenomic library of the slug’s gut microbiota. This library comprises proteins with putative functions classified under different CAZyme families (Table 1). The translated nucleotide sequence of Gene_id_40363 returned a conserved domain function description for putative esterase and showed 92.7% similarity with an uncharacterized esterase from *Buttiauxella agrestis* (Max score: 420 and E-value: 7 × 10^−148^). The sequence of Gene_id_40363 contained the predicted SGNH hydrolase-type esterase domain and belonged to the GFSQ-like lipase abhydrolase family (abhydrolase_2; Pfam domain PF02230; Figure 2).

**Figure 3:**
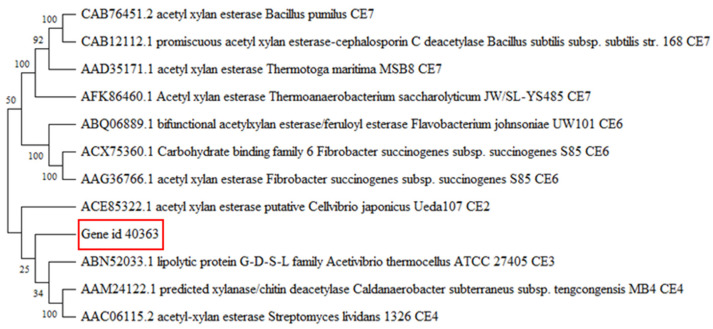
Phylogenetic tree. The nucleotide sequence of Gene_id_40363 was used to construct a phylogenetic tree with characterised proteins of CE family 1–7 with 3D models available in PDB. A Neighbour-Joining phylogenetic tree was constructed in MEGAX with 1000 bootstrap replicates. Proteins are identified with their GenBank number and described with their function(s) and CAZy classification.

The CE1 family contains over 5000 protein entries and covers activities including acetyl xylan esterase, cinnamoyl esterase, feruloyl esterase, carboxylesterase, S-formylglutathione hydrolase, diacylglycerol *O*-acyltransferase, and trehalose 6-*O*-mycolyltransferase and several other esterases, such as poly (β-hydroxybutyrate) depolymerase. However, only 11 proteins (10 from eukaryote sources and 1 from bacteria sources) have been characterised so far in this family. Homology models developed in I-Tasser using the closest structural similarity based on the threading program returned the α/β fold model of the abhydrolase protein family. The homology model features 5 α-helices and 6 β-sheets, and the catalytic triad was observed at positions S111, D159, and H191. The homology model of Gene_id_40363 showed the absence of lid structure, but three loop structures were observed around the active site region (Figure 2). We accessed relatedness with members of other CE families. Gene_id_40363 shares low sequence identity with characterised proteins in the CE family that have a 3D structure model available in PDB (Figure 3). 

### 2.2. Recombinant Protein and Activity Assay

**Figure 4:**
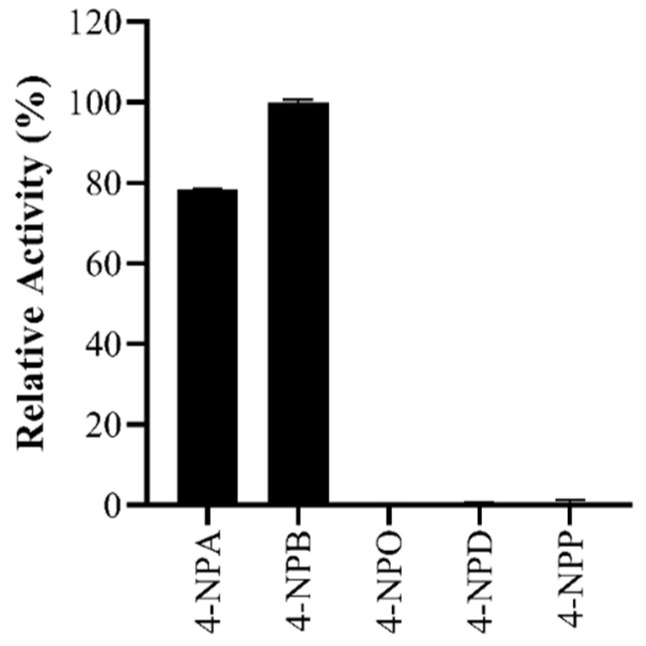
Activity assay. BaAXE was assayed using various acyl chain esters (C2–C16). 4-NPA—4-nitrophenyl acetate; 4-NPB—4-nitrophenyl butyrate; 4-NPO—4-nitrophenyl octanoate; 4-NPD—4-nitrophenyl decanoate; 4-NPP—4-nitrophenyl palmitate. The assay reaction was carried out with a 1 mM concentration of each substrate, and the reaction proceeded for 20 min at 40 °C. Absorbance readings were taken at 410 nm at 1 min intervals. Each bar represents activity on each substrate, *n* = 3 and the error bar corresponds to the standard error of the mean (SEM).

Recombinant protein of Gene_id_40363 (BaAXE) was expressed in *E. coli* BL21 (DE3) with a C-terminal 6x His-tag to enhance detection and purification. The recombinant protein showed a molecular weight of 27 kDa estimated by SDS-PAGE and confirmed with Western blot detection. The observed size corresponds to the theoretical value estimated by ProtParam hosted on Expasy. The purified protein was eluted with buffers containing 105 mM and 120 mM imidazole (Supplementary Data Figure S1). BaAXE predicted as an esterase was active on acetate substrates—4-nitrophenyl acetate (specific activity: 78.12 U/mg) and 4-methylumbelliferyl acetate (specific activity: 2.49 U/mg). Furthermore, BaAXE was active on 4-nitrophenyl butyrate (specific activity: 99.73 U/mg) but showed no clear activity on longer-chain acyl chain esters (C8–C16; Figure 4).

### 2.3. Biochemical Properties 

**Figure 5:**
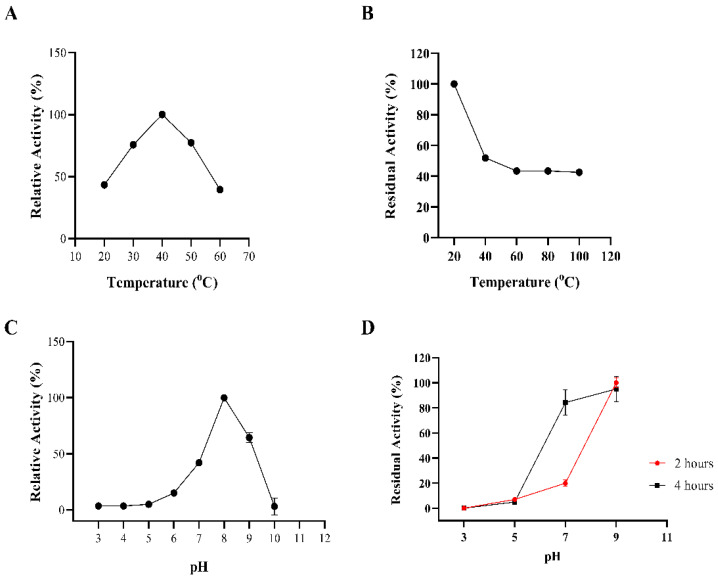
Biochemical properties. The biochemical properties of BaAXE were determined in assays with 4-NPA. (**A**) The optimal temperature was determined in assays performed at different temperatures: 20 °C, 30 °C, 40 °C, 50 °C, and 60 °C. (**B**) Thermostability assay determined after incubating the enzyme for 2 h at different temperatures. (**C**) The optimal pH was determined in assays performed at different pHs at the optimal temperature. (**D**) pH stability was determined after incubation of the enzyme for 4 h at 20 °C. After 2 h of incubation, residual activity was determined, and after 4 h, the residual activity was determined. Absorbance readings were measured at 410 nm. Assay without the enzyme was used as blank. Assays were performed in triplicate, and error bars correspond to SEM.

The biochemical properties of BaAXE were investigated in assays with 4-NPA as the substrate. BaAXE showed optimal activity at pH 8 and 40 °C. The km, kcat, and kcat/km (catalytic efficiency) values were calculated as 0.43 mM, 122.4 s^−1^, and 282 mM^−1^ s^−1^, respectively. A thermostability assay showed that the BaAXE retained around 40% activity after incubating the enzyme for 2 h at 40–100 °C but showed no clear activity at acidic pHs. At pH 7 and 9, BaAXE retained over 80% activity after incubating the enzyme for 4 h (Figure 5). 

**Figure 6:**
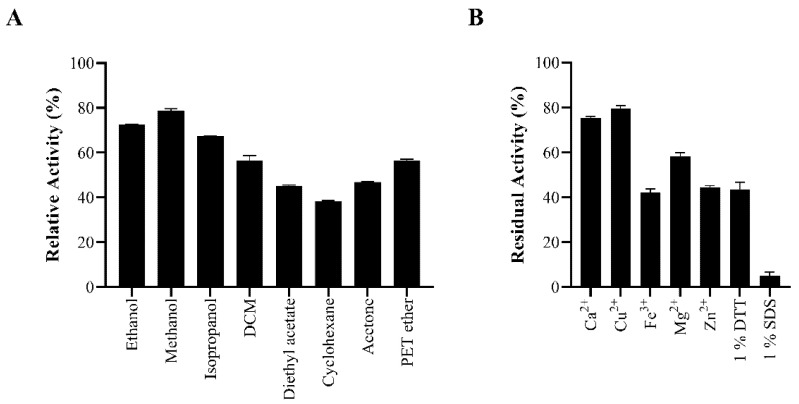
Inhibition by organic solvents and additives. The enzyme was assayed in the presence of (**A**) 20% selected organic solvent and (**B**) 1 mM metal ion, 1% DTT, and 1% SDS. Reactions proceeded for 10 min, and absorbance reading was measured at 410 nm. Assays were performed in triplicate, and error bars correspond to SEM. Relative activity was determined relative to a control reaction lacking any additive or organic solvent. DCM—dichloromethane, PET—petroleum ether.

We investigated the effect of organic solvents and additives on enzyme activity. BaAXE was not severely impacted by organic solvents at 20% (*v*/*v*) retaining over 60% activity in most of the solvents tested after a 17 h incubation. None of the metal ions tested severely impacted its activity, as the enzyme retained over 40% activity after incubation in a 1 mM concentration of the metal salt. The activity of the enzyme was severely impacted when incubated with 1% SDS, retaining only 5% activity after incubation (Figure 6). 

### 2.4. Functional Characterisation

**Figure 7:**
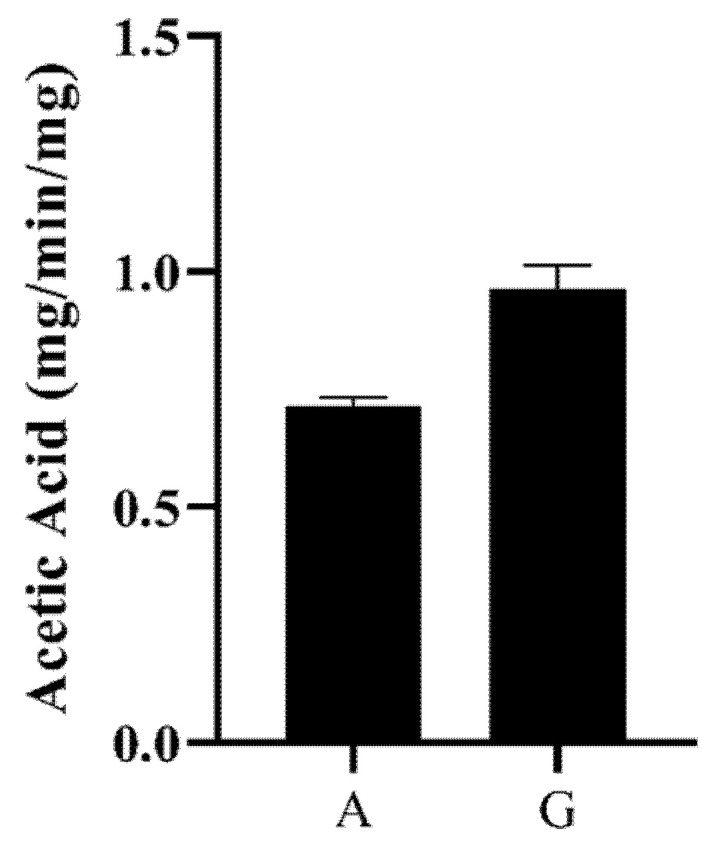
Acetic acid release. The activity of the enzyme on acetylated monosaccharides and xylooligosaccharides was determined by the hydrolytic release of acetic acid from these substrates. Acetic acid release was measured with GC–MS after incubating the enzyme with birchwood xylan (partially acetylated) (A) and β-d-glucosepentaacetate (G). The acetic acid release was estimated from an acetic acid standard plot.

BaAXE was assayed on various substrates to investigate the functional characterization of the enzyme. The enzyme showed no activity on the α-l-arabinofuranosidase substrate—4-nitrophenyl arabinofuranoside, and no activity was detected on ferulic esterase substrates—chromogenic acid and benzyl cinnamate. Furthermore, the enzyme was tested for methylesterase activity and showed no activity on pectin substrate (poly-d-galacturonic acid methyl ester). However, the enzyme released acetic acid from acetylated monosaccharaides and acetylated xylooligosaccharides (Figure 7). 

## 3. Discussion

The recalcitrance of biomass is a major obstacle to harnessing its full potential. Reducing acetylation is a promising approach to reducing recalcitrancy in LCB, allowing for the sustainable production of chemicals and fuels [38]. Here, we reported the characterisation of a novel acetyl xylan esterase (designated as BaAXE), which was previously annotated as Gene_id_40363 in the metagenomic library of the gut microbiota of the common black slug. Carboxylesterases are enzymes distinguished by the α/β-hydrolase fold containing the Ser-His-Asp catalytic triad with serine residue as the nucleophile. Acetyl xylan esterases are carboxylesterases known to hydrolyse ester linkages in acetylated xylan to liberate acetic acid. Hence, they are important auxiliary enzyme components for the degradation of LCB [39].

The translated nucleotide sequence matched functions described as predicted hydrolase and shared 92% sequence similarity with an uncharacterised esterase from *Buttiauxella agrestis*. BaAXE was shown through multiple sequence alignment to contain the GFSQG motif around the active site serine. Acetyl xylan esterases are reported as serine-type esterases but might differ in the residues surrounding the active site serine [40]. However, BaAXE shares low sequence similarity among characterised carbohydrate esterase (CE) with 3D structures submitted in PDB but was assigned to the abhydrolase_2 (pfam02230) family, consisting of both phospholipases and carboxylesterases that have broad substrate specificity and are structurally related to the α/beta hydrolases (pfam00561). Furthermore, in the metagenomic library in which it was identified, it was designated as a member of the CE1 family, which consists of over 5000 entries and 11 characterised proteins. Characterized proteins in this family are AXEs from fungal sources, and no AXE from bacteria has been reported in this family. Hence, BaAXE is likely the first characterized AXE from the CE1 family. Enzymes from bacterial sources have several advantages over enzymes from fungal sources; for example, bacteria exist almost everywhere, are highly adaptable, and are easier to genetically modify [8]. Hydrolases of utmost importance are classed as lipases (triacylglycerol hydrolases) and esterases (carboxyl ester hydrolases). Carboxylester hydrolases, such as pectin methylesterases, acetyl xylan esterase, and feruloyl esterase, are known to act on plant cell wall polysaccharides [41]. The homology three-dimensional (3D) model of BaAXE confirms the presence of the canonical α/β hydrolase fold of the abhydrolase family and the presence of the active site residues S111, D159, and H191, also referred to as the catalytic triad [42]. The crystal structure of a carboxylesterase from *Pseudomonas fluorescens* showed the α/β hydrolase fold containing the Ser-His-Asp catalytic triad, and the active site clefts were relatively open—solvent-exposed—similar to our deduction from the 3D homology model of BaAXE [43]. Lipases are distinguished from esterase by the occurrence of interfacial activation, which is due to the lid domain covering the active sites of lipases. This domain is lacking in BaAXE, as observed in the modelled structure; hence, BaAXE is likely to be identified as an esterase [44,45].

The recombinant enzyme BaAXE showed clear activity toward C2 and C4 acyl chain substrates and was inactive on longer chain acyl chains (C > 6), confirming its classification as an esterase and not a lipase. Carboxylesterases are known to catalyse the hydrolysis of short-chain aliphatic and aromatic esters with broad specificity [46]. Furthermore, BaAXE did not exhibit FAE activity or methyl esterase activity but released acetic acid from acetylated monosaccharides and xylooligosaccharides, confirming its acetyl xylan esterase classification. An acetyl xylan esterase from *Aspergillus oryzae*, designated as AoAXEC, released acetic acid from wheat arabinoxylan but was inactive on methyl esters of ferulic, ρ-coumaric, caffeic, or sinapic acids [28]. To investigate the biochemical activity of BaAXE, we assayed the pH and temperature profile of the enzyme using 4-nitrophenyl acetate as the substrate. The recombinant enzyme showed optimum activity at pH 8.0 and 40 °C which is similar to previously characterised acetyl xylan esterases (Table 2). Most notably, the enzyme showed interesting thermostability, retaining more than 40% residual activity after incubation at 40 °C–100 °C for 2 h. Most characterised AXEs did not show activity at acidic pHs (2–5) and showed an optimal pH in an alkaline range (7.5–8.5). BaAXE retained over 80% residual activity after incubation at pH 7–9 for 4 h. This is an interesting characteristic, as stability in alkaline environments is of interest in several bio-industrial applications, especially in direct applications in alkaline-pretreated biomass [47]. A novel acetyl xylan esterase from *Flavobacterium johnsoniae* (FjoAcXE) showed similar biochemical properties to BaAXE; however, FjoAcXE had no residual activity after 5 min of incubation at 60 °C [35]. Most of the characterised AXEs are thermolabile, especially when incubated at over 60 °C (Table 2). BaAXE obeyed the classical Michaelis–Menten kinetics, reporting a Km value of 0.4 mM and Kcat value of 122.4 s^−1^. This property of BaAXE further supports its classification as an esterase, as lipases do not obey the classical Michaelis–Menten kinetics and require only a minimum substrate concentration before activity is observed [42].

We reported the biochemical and functional characterisation of a novel acetyl xylan esterase that was not previously classified in the six families of AXEs in the ESTHER database [28]. However, the functional characterisation of BaAXE reported here strongly recommends its assignment into one of the nine AXE families in the CAZy database [48]. Harnessing the unique properties of BaAXE, most notably its stability in a wide range of temperatures and stability at an alkaline pH, would benefit industrial applications. BaAXE liberated acetic acid from xylan (birchwood, partially acetylated) and β-d-glucopyranose pentaacetate, indicating that BaAXE is capable of deacetylation at various acetyl substitutions. Cell wall polysaccharides are either mono- or di-acetylated, and the positions of these acetylations vary among cell wall polysaccharides. Acetylation in xylan usually occurs at position O-2 or O-3, and a xylopyranose may be acetylated at position O-3 [49]. It would be interesting to investigate the synergetic activity of BaAXE with xylanases. The simultaneous treatment of AXE from *Lactobacillus antri* with xylanase showed a 1.44-fold increase in the degradation of beechwood xylan compared with xylanase treatment alone [40]. Furthermore, carboxylesterases have been implicated in the biosynthesis of compounds and the resolution of racemic mixtures [41]. It would be interesting to investigate the stereospecific synthetic ability of BaAXE, notably the synthesis of platform chemicals (such as muconic acid) from lignocellulose substrates [50], as well as the deacetylation prospects of BaAXE on non-xylooligosaccharide substrates, such as polyvinyl acetate [51]. CE1 enzymes have been described with functions relating to the degradation of natural polyesters, such as poly 3-hydroxybutyrate (PHB) depolymerase, and esterases have been implicated as promising candidates for the degradation of polyester-based plastics [52]. It would be informative to explore other applications of this enzyme, as BaAXE promises to be a promising candidate in developing an enzyme consortium targeting the complete degradation of lignocellulose biomass and the development of novel chemicals.

**Table 2:** Characteristics of acetyl xylan esterases.

Protein Designation	Source Organism	Temp. Opt.	pH Opt.	Temp. Stability	Reference
axeA	*Aspergillus awamori*	-	7	Enzyme activity decreased at temperatures higher than 40 °C.	[53]
axeA	*Aspergillus ficuum*	40 °C	7	Thermal stability decreased at temperatures above 40 °C.	[54]
rAoAXE	*Aspergillus oryzae*	45 °C	6	Unstable at 40 degrees Celsius, with a half-life of less than 60 min at 40 °C and 10 min at 50 °C	[55]
AXE	*Bacillus pumilus* PS213	55 °C	8	Stable at 50 °C but rapidly inactivated at a temperature higher than 60 °C, with a half-life of about 1 h at this temperature	[56]
TM0077	*Thermotoga maritima*	100 °C	7.5	Unstable at 100 °C with a half-life of <5 min.	[57]
AxeA	*Thermotoga maritima*	90 °C	6.5	Retained over 60% residual activity after 8 h incubation at 98 °C	[58]
AXE	*Ochrovirga pacifica*	50 °C	8	Maintained over 70% residual activity after incubation at 55 °C for 120 min	[25]
rAoAXEC	*Aspergillus oryzae*	50 °C	7	Stable up to 50 °C, with a half-life of approximately 2 h at 50 °C and 40 min at 60 °C	[28]
LaAXE	*Lactobacillus antri*	50 °C	7	At 70 °C, the residual activity decreased to 48.4%	[40]
AXE1	*Thermoanaerobacterium* sp.	80 °C	7	At 75 °C, the enzyme showed a half-life of 1 h.	[59]

## 4. Materials and Methods

### 4.1. Materials

The slug gut metagenome DNA was provided by Dr Natalie Ferry, University of Salford, Manchester, United Kingdom. The substrates—4-nitrophenyl acetate, 4-nitrophenyl butyrate, 4-nitrophenyl octanoate, 4-nitrophenyl decanoate, and 4-nitrophenyl palmitate, β-D-glucose pentaacetate, pectin, chlorogenic acid, benzyl cinnamate, and 4-methylumbelliferyl acetate—were purchased from Sigma-Aldrich (Gillingham, United Kingdom). Xylan (birchwood, partially acetylated) was purchased from Megazyme (Wicklow, Ireland). Other chemicals were of molecular grade and purchased from Sigma-Aldrich (Gillingham, United Kingdom). Primers for PCR were synthesised and sequenced by Eurofins (Konstanz, Germany). BL21 and TOP10 competent cells were purchased from ThermoFisher Scientific (Leicester, United Kingdom).

### 4.2. Methods 

#### 4.2.1. Bioinformatics 

The output of the functional metagenomic screening of the slug gut microbiota was in the format of a translated nucleotide sequence in FASTA format (http://www.ebi.ac.uk/ena/data/view/PRJEB21599, accessed on 25 January 2022). The amino acid sequence identified in the metagenomic library as Gene_id_40363 was selected for characterisation. To verify the putative protein function, the amino acid sequence was used to query the non-redundant protein sequence database using the default algorithm for the BLASTp program hosted on the NCBI website (https://www.ncbi.nlm.nih.gov/, accessed on 10 March 2022). Amino acid sequences selected for phylogenetic tree construction were aligned with MUSCLE (Codons) available in MEGA software (version X) [60] using the default algorithm, and the UPGMA (unweighted pair group method) was selected as the cluster method. The amino acid sequences of proteins that had been previously functionally characterised with available 3D models in PDB (as reported in the CAZy database) were selected for constructing the phylogenetic tree. Tree construction and phylogenetic analysis were performed in MEGA software (version X) [60] with the neighbour-joining method and the Poisson model. Trees were validated with the bootstrapping method using 1000 Bootstrap replications [61]. 

The annotation of protein features was achieved with Multiple Sequence Alignment (MSA). Multiple sequence alignment was performed with ClustalO hosted on the EMBL-EBI website using the default algorithm (https://www.ebi.ac.uk/Tools/, accessed on 10 March 2022). The amino acid sequence of Gene_id_40363 was aligned with the amino acid sequence of the top hit protein when queried against the swissprot database and closely related proteins identified from the constructed phylogenetic tree [25]. The annotation and display of the sequence alignment were performed in GeneDoc (version 2.7). The molecular weight and isoelectric point (pI) of proteins were determined using the ProtParam tool available on ExPASy (https://web.expasy.org/protparam/, accessed on 15 March 2022). The three-dimensional (3D) model of Gene_id_40363 was generated with homology modelling performed in Iterative Threading ASSEmbly Refinement (I-TASSER; https://zhanglab.ccmb.med.umich.edu/I-TASSER, accessed on 15 March 2022) using multiple sequences of crystalised proteins as a template [61,62]. The modelled structure was validated based on the C-score (confidence score), TM-score (template modelling score), and RMSD (root-mean-square deviation), as previously described [62]. The final protein model was visualised and annotated in PyMOL version 2.0 (Schrodinger New York, NY, USA). 

#### 4.2.2. Amplification, Cloning, and Bacterial Transformation 

Gene_id_40363 was amplified from the slug’s gut metagenomic DNA using gene-specific primers—FP: CACCATGAAACATGACCAC, RP: GCCTCATCGAAATAGTGC—and the following PCR conditions: initial denaturation at 98 °C for 1 min; 35 cycles of denaturation at 98 °C for 40 s, an annealing temperature of 61 °C for 30 s, and extension for 19 s at 72 °C; and a final extension time of 10 min. PCR amplification was conducted with a high-fidelity thermostable DNA polymerase Q5 (NEB, Hitchin, UK) in a 25 µL reaction containing 12.5 µL of Q5 Hi-Fidelity 2X Master Mix, 1.25 µL of (10 µM forward and 10 µM reverse primers), and ~60 ng of template DNA.

**Figure 8:**
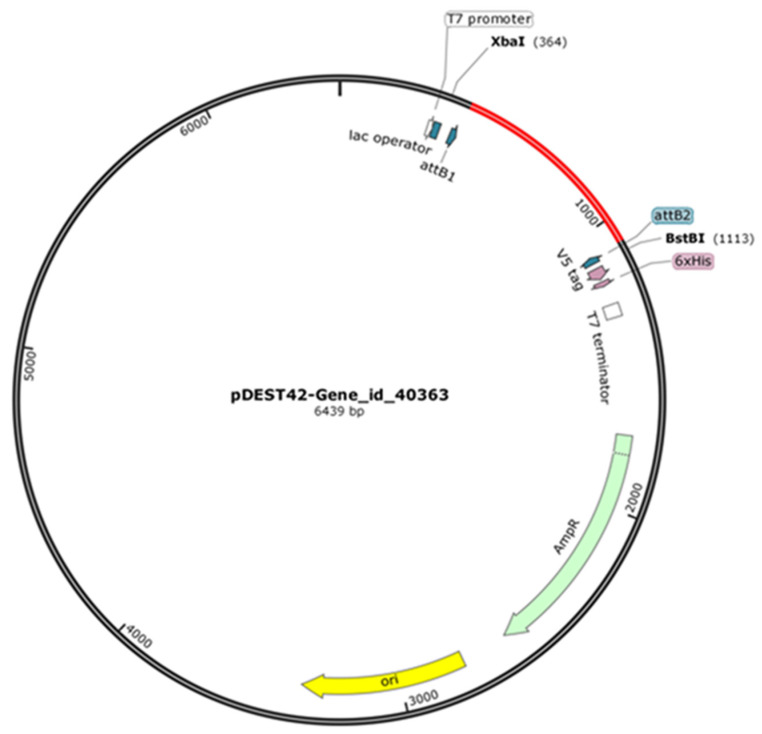
Expression vector construct. The expression plasmid with Gene_id_40363 ligated was modelled in SnapGene (Dotmatics San Diego, USA). Position of gene insert is indicated by the red segment, and selected plasmid features are also annotated.

The PCR-amplified product (654 bp) was cloned into an entry vector pENTR/SD/D/TOPO (ThermoFischer Scientific Leicester, UK) following the manufacturer’s protocol. To generate the recombinant expression vector (Figure 8), pENTR: Gene_id_40363 was recombined with the destination vector pDEST42 (ThermoFischer Scientific, Leicester, UK) following the manufacturer’s protocol. The recombinant expression vector was used to transform *Escherichia coli* (*E. coli*) DE3 cells. The desired transformant was screened with restriction digest and sequencing.

#### 4.2.3. Protein Expression and Purification

Recombinant plasmid (pDEST42-Gene_id_40363) was used to transform *E. coli* BL21 (DE3) cells for recombinant protein expression with IPTG induction. Transformed cells were grown to an OD600 of 0.5 before induction with 0.6 mM IPTG. Protein expression was allowed for 6 h at 30 °C with shaking at 225× *g*. Cells pellets were harvested by centrifugation at 100× *g* for 10 min at 4 °C. Cell pellets were lysed in a lysis buffer (50 mM sodium phosphate, 150 mM NaCl, 0.1% triton x-100, and 10 mM imidazole) for 30 min at 4 °C. After cell lysis, the soluble fraction was recovered by centrifugation at 4600 rpm for 15 min at 4 °C. The recombinant His-tagged protein was purified with a His GraviTrap TALON gravity flow column charged with cobalt ions, following the manufacturer’s protocol (Cytiva, Sheffield, United Kingdom). The elution step was performed with a gradient imidazole concentration (30 mM–150 mM imidazole). Imidazole and excess salt were dialysed out using SnakeSkin dialysis tubing following the manufacturer’s protocol (ThermoFisher Scientific Leicester, UK). The purified protein was concentrated using the Amicon ultra centrifugal filter unit (10 kDa cut-off, Millipore, Watford, UK).

#### 4.2.4. Enzyme Assay and Biochemical Properties 

The purified and concentrated recombinant protein was assayed for activity using synthetic substrates. Esterase activity was determined spectrophotometrically with 4-nitrophenyl acetate (4-NPA) as the substrate. The assay reaction contained 50 mM sodium phosphate, pH 7.5; 150 mM NaCl; and 0.01% triton x-100. A stock solution (250 mM) of the substrate was prepared in dichloromethane, and a final concentration of 1 mM substrate in 100 µL was used in the assay. The enzyme reaction was initiated by adding a properly diluted enzyme solution (3–5 μg). The enzyme reaction was performed at 40 °C, and the absorbance measurement was monitored every minute for 15 min at 410 nm using a Varioskan LUX Multimode Microplate Reader (ThermoFischer Scientific, Leicester, UK). The background hydrolysis of the substrate was subtracted by performing a blank reaction without the enzyme. One unit of enzymatic activity was defined as the amount of the protein releasing 1 µmol of 4-nitrophenol per minute at the assay conditions described.

#### 4.2.5. Biochemical Properties

The enzyme’s temperature profile was determined in an assay with a 1 mM concentration of 4-NPA as described above at varying temperatures. The pH profile was determined at the optimum temperature under various experimental pHs. pH 3 and 4 were prepared in sodium citrate buffer, pH 5 and 6 were prepared in Hepes buffer, pH 7 and 8 were prepared in sodium phosphate buffer, and pH 9 and 10 were prepared in glycine NaOH buffer. The assay was performed for 10 min, and activity was expressed as the percentage relative activity with respect to the maximum activity. The enzyme kinetics assay was carried out at pH 8 using 4-NPA at concentrations from 0.1 mM to 2 mM. The kinetic parameters—Km and Vmax—were calculated by a nonlinear regression fit based on the Michaelis–Menten model using GraphPad Prism 9 [62,63].

#### 4.2.6. Thermostability 

The thermal stability and pH stability were determined as described by Razeq et al. (2018). For temperature stability, 4 µg of enzyme in 50 mM sodium phosphate (pH 7.0)—final volume of 40 µL—was incubated at 20 °C, 30 °C, 40 °C, and 50 °C for 2 h. After incubation, the enzyme was ice-cooled for 5 min before the residual activity was determined in the standard assay with 4-NPA. 

#### 4.2.7. Effect of Organic Solvents and Additives

The effect of organic solvents on enzyme activity was assayed by incubating 4 µg of the enzyme in 20% concentrations of various organic solvents for 17 h at 4 °C. The residual activity was determined in a standard assay with 1 mM 4-NPA. Residual activity is expressed as a percentage relative to a control (assay without organic solvent addition) set at 100%.

The effect of additives, such as detergents and divalent ions, was determined. The effect of metal ions was determined in reactions with 1 mM 4-NPA as the substrate. The reaction mixture contained a 1mM concentration of the following metal ions: Ca^2+^, Co^2+^, Cu^2+^, Fe^3+^, Mg^2+^, and Zn^2+^ (all as chloride salts). The effect of other additives was determined by the addition of 20 mM EDTA, 1% Triton x-100, 1% tween-20, 1% (*w*/*v*) dithiothreitol (DTT), and 1% (*w*/*v*) SDS. The residual activity was determined in a standard assay with 1 mM 4-NPA.

#### 4.2.8. Acyl Chain Substrate

The substrate specificity of Gene_id_40363 was determined by assaying its activity on various 4-NP alkyl esters—4-nitrophenyl acetate, 4-nitrophenyl butyrate (4-NPB), 4-nitrophenyl octanoate (4-NPO), 4-nitrophenyl decanoate, and 4-nitrophenyl palmitate (4-NPP). The final concentration of the substrate in each assay was 1 mM. Reactions were performed in 50 mM sodium phosphate, as described in Section 4.2.4, and initiated by adding 4 µg of protein. The reaction was allowed for 15 min, and the reaction without the enzyme was used as a blank.

#### 4.2.9. 4-Methylumberiferyl Acetate (4-MUA)

The activity of Gene_id_40363 was assayed on 4-methylumberiferyl acetate (4-MUA). The reaction mixture contained 50 mM sodium phosphate (pH 8.0) and 1 mM 4-MUA dissolved in DMSO. The assay was initiated by adding 4 µg of protein in a total reaction volume of 200 µL. The release of 4-umberiferinone was monitored by reading the fluorescence (excitation/emission 340/520) using a Varioskan LUX Multimode Microplate Reader (ThermoFischer Scientific, Leicester, UK). The assay reaction was incubated at 40 °C, and readings were taken every 1 min for 30 min. Reaction without the enzyme was used as blank. One unit of enzymatic activity was defined as the amount of the protein releasing 1 µmol of 4-methylumbelliferone per minute at the assay conditions described.

#### 4.2.10. Hydrocinnamate Substrates

The activity of Gene_id_40363 towards hydrocinnamate substrates (chlorogenic acid and Benzyl cinnamate) was assayed as previously described [47]. The substrates were solved in DMSO, and the assay was initiated with 4 µg of purified protein. The decrease in substrate concentration was spectrophotometrically quantified following absorbance at 340 nm. The assay was quantified with reference to a standard curve. One unit of enzyme is defined as the amount of enzyme releasing 1 µg of substrate in 1 min under the assay conditions.

#### 4.2.11. Acetic Acid Release from Acetylated Substrates

**Figure 9:**
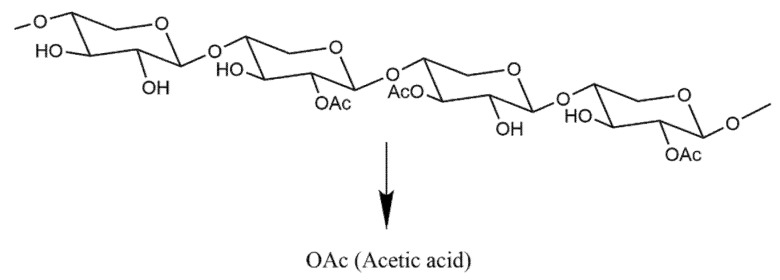
Acetylated xylan. The release of acetic acid is achieved by the action of acetyl xylan esterase on acetylated xylan.

The activity of Gene_id_40363 was assayed on acetylated xylan (Figure 9) and β-D-glucose pentaacetate. Acetylated xylan (1%) or β-d-glucose pentaacetate (0.1%) was incubated with 4 µg of enzyme at 40 °C for 10 min. After incubation, the reaction was equilibrated 1:1 (*v*/*v*) with acetonitrile. Acetic acid release was analysed with GC–MS (59778B GC/MSD, 8860 GC system, Agilent technologies, Cheadle, UK). The samples were run for 20 min using an Agilent 19091s-433UI HP-5ms Ultra Inert (30 m × 250 µm × 0.25 µm) column. The MS parameters were set as follows: ion source—EI, source temperature—230 °C, quad temperature–150 °C, fixed electron energy—70 eV, solvent delay—2 min. The acquisition was set to scan a mass range of 40–200. The method report for the MS run is reported in the Supplementary Materials section (Figure S2). Reaction mixtures without the enzymes were used as a negative control. Acetic acid content was quantified using the abundance data from various concentrations of acetic acid standards.

## Figures and Tables

**Table 1:** Hemicellulases screened from the metagenomic library of the slug’s gut microbiota.

CAZy Family	Activity *	No. of Genes
CE 1	Acetyl xylan esterase	120
CE 10	Esterase	38
GH 43	β-Xylosidase, α-L-arabinofuranosidase, xylanase and arabinanase	55
GH 5	Endo-β-1,4-xylanase, β-mannosidase	15
GH 12	Endoglucanase, xyloglucan hydrolase	13
GH 16	Xyloglucanse, endo-1,3-β-glucanase	117
GH 10	Endo-1,4-β-xylanase, endo-1,3-β-xylanase	16
GH 67	α-Glucuronidase, xylan α-1,2-glucuronidase	1
GH 51	Endoglucanase, endo-β-1,4-xylanase, β-xylosidase, α-L-arabinofuranosidase	3
CE 6 and CE 7	Acetyl xylan esterase	5
GH 8	Endo-1,4-β-xylanase	11
GH 38	α-Mannosidase	39
GH 39	β-Xylosidase	279

* Protein family function as described on CAZy.org.

## Data Availability

Not applicable.

## Supplementary Materials

The following supporting information can be downloaded at: https://www.mdpi.com/article/10.3390/molecules27092999/s1, Figure S1: Purification of Gene_id_40363; Figure S2: MS parameter report.

## Sample Availability

The plasmid construct used in this study is available from H.M.

## Abbreviations

LCB—lignocellulose biomass; AXE—acetyl xylan esterase—GC–MS—gas chromatography–mass spectrometry; 4-NPA—4-nitrophenyl acetate; 4-NPB—4-nitrophenyl butyrate; 4-NPO—4-nitrophenyl octanoate; 4-NPD—4-nitrophenyl decanoate; 4-NPP—4-nitrophenyl palmitate; CE—carbohydrate esterase; GH—glycosyl hydrolase.

## References

[1] Kumar R., Strezov V., Weldekidan H., He J., Singh S., Kan T., Dastjerdi B. (2020). Lignocellulose Biomass Pyrolysis for Bio-Oil Production: A Review of Biomass Pre-Treatment Methods for Production of Drop-in Fuels. Renew. Sustain. Energy Rev..

[2] Aghbashlo M., Khounani Z., Hosseinzadeh-Bandbafha H., Gupta V.K., Amiri H., Lam S.S., Morosuk T., Tabatabaei M. (2021). Exergoenvironmental Analysis of Bioenergy Systems: A Comprehensive Review. Renew. Sustain. Energy Rev..

[3] Rittmann B.E. (2008). Opportunities for Renewable Bioenergy Using Microorganisms. Biotechnol. Bioeng..

[4] Lorenci Woiciechowski A., Dalmas Neto C.J., Porto de Souza Vandenberghe L., de Carvalho Neto D.P., Novak Sydney A.C., Letti L.A.J., Karp S.G., Zevallos Torres L.A., Soccol C.R. (2020). Lignocellulosic Biomass: Acid and Alkaline Pretreatments and Their Effects on Biomass Recalcitrance—Conventional Processing and Recent Advances. Bioresour. Technol..

[5] Abraham A., Mathew A.K., Park H., Choi O., Sindhu R., Parameswaran B., Pandey A., Park J.H., Sang B.I. (2020). Pretreatment Strategies for Enhanced Biogas Production from Lignocellulosic Biomass. Bioresour. Technol..

[6] Sindhu R., Binod P., Pandey A. (2016). Biological Pretreatment of Lignocellulosic Biomass—An Overview. Bioresour. Technol..

[7] Hazeena S.H., Sindhu R., Pandey A., Binod P. (2020). Lignocellulosic Bio-Refinery Approach for Microbial 2,3-Butanediol Production. Bioresour. Technol..

[8] Kumar B., Bhardwaj N., Agrawal K., Chaturvedi V., Verma P. (2020). Current Perspective on Pretreatment Technologies Using Lignocellulosic Biomass: An Emerging Biorefinery Concept. Fuel Process. Technol..

[9] Rebello S., Anoopkumar A.N., Aneesh E.M., Sindhu R., Binod P., Pandey A. (2020). Sustainability and Life Cycle Assessments of Lignocellulosic and Algal Pretreatments. Bioresour. Technol..

[10] Raghavi S., Sindhu R., Binod P., Gnansounou E., Pandey A. (2016). Development of a Novel Sequential Pretreatment Strategy for the Production of Bioethanol from Sugarcane Trash. Bioresour. Technol..

[11] Sindhu R., Kuttiraja M., Prabisha T.P., Binod P., Sukumaran R.K., Pandey A. (2016). Development of a Combined Pretreatment and Hydrolysis Strategy of Rice Straw for the Production of Bioethanol and Biopolymer. Bioresour. Technol..

[12] Xu J., Zhao X., Yao Q., Zong W., Dai S., Deng Z., Liu S., Yun J., Yang X., Li H. (2021). Cloning, Characterization of a Novel Acetyl Xylan Esterase, and Its Potential Application on Wheat Straw Utilization. All Life.

[13] Farhat W., Venditti R., Ayoub A., Prochazka F., Fernández-de-Alba C., Mignard N., Taha M., Becquart F. (2018). Towards Thermoplastic Hemicellulose: Chemistry and Characteristics of Poly-(ε-Caprolactone) Grafting onto Hemicellulose Backbones. Mater. Des..

[14] Huang L.Z., Ma M.G., Ji X.X., Choi S.E., Si C. (2021). Recent Developments and Applications of Hemicellulose from Wheat Straw: A Review. Front. Bioeng. Biotechnol..

[15] Ahmad N., Tayyeb D., Ali I., Alruwaili N.K., Ahmad W., ur Rehman A., Khan A.H., Iqbal M.S. (2020). Development and Characterization of Hemicellulose-Based Films for Antibacterial Wound-Dressing Application. Polymers.

[16] Hameleers L., Penttinen L., Ikonen M., Jaillot L., Fauré R., Terrapon N., Deuss P.J., Hakulinen N., Master E.R., Jurak E. (2021). Polysaccharide Utilization Loci-Driven Enzyme Discovery Reveals BD-FAE: A Bifunctional Feruloyl and Acetyl Xylan Esterase Active on Complex Natural Xylans. Biotechnol. Biofuels.

[17] Marasinghe S.D., Jo E., Hettiarachchi S.A., Lee Y., Eom T.Y., Gang Y., Kang Y.H., Oh C. (2021). Characterization of Glycoside Hydrolase Family 11 Xylanase from *Streptomyces* Sp. Strain J103; Its Synergetic Effect with Acetyl Xylan Esterase and Enhancement of Enzymatic Hydrolysis of Lignocellulosic Biomass. Microb. Cell Factories.

[18] Binod P., Gnansounou E., Sindhu R., Pandey A. (2019). Enzymes for Second Generation Biofuels: Recent Developments and Future Perspectives. Bioresour. Technol. Rep..

[19] Shen D.K., Gu S., Bridgwater A.V. (2010). The Thermal Performance of the Polysaccharides Extracted from Hardwood: Cellulose and Hemicellulose. Carbohydr. Polym..

[20] Park S.H., Yoo W., Lee C.W., Jeong C.S., Shin S.C., Kim H.W., Park H., Kim K.K., Kim T.D., Lee J.H. (2018). Crystal Structure and Functional Characterization of a Cold-Active Acetyl Xylan Esterase (PbAcE) from Psychrophilic Soil Microbe *Paenibacillus* sp.. PLoS ONE.

[21] Joshi N., Sharma M., Singh S.P. (2020). Characterization of a Novel Xylanase from an Extreme Temperature Hot Spring Metagenome for Xylooligosaccharide Production. Appl. Microbiol. Biotechnol..

[22] Houfani A.A., Anders N., Spiess A.C., Baldrian P., Benallaoua S. (2020). Insights from Enzymatic Degradation of Cellulose and Hemicellulose to Fermentable Sugars—A Review. Biomass Bioenergy.

[23] Ohta K., Fujii S., Higashida C. (2013). Characterization of a Glycoside Hydrolase Family-51 α-l-Arabinofuranosidase Gene from Aureobasidium Pullulans ATCC 20524 and Its Encoded Product. J. Biosci. Bioeng..

[24] Wang L., Han X., Wang Y., Wei X., Liu S., Shao S., Yang S., Sun L., Xin F. (2021). Rational Design for Broadened Substrate Specificity and Enhanced Activity of a Novel Acetyl Xylan Esterase from Bacteroides Thetaiotaomicron. J. Agric. Food Chem..

[25] Hettiarachchi S.A., Kwon Y.K., Lee Y., Jo E., Eom T.Y., Kang Y.H., Kang D.H., de Zoysa M., Marasinghe S.D., Oh C. (2019). Characterization of an Acetyl Xylan Esterase from the Marine Bacterium Ochrovirga Pacifica and Its Synergism with Xylanase on Beechwood Xylan. Microb. Cell Fact..

[26] Puchart V., Gjermansen M., Mastihubová M., Mørkeberg Krogh K.B.R., Biely P. (2020). Positional Specificity of Flavobacterium Johnsoniae Acetylxylan Esterase and Acetyl Group Migration on Xylan Main Chain. Carbohydr. Polym..

[27] Zhang Y., Yang H., Yu X., Kong H., Chen J., Luo H., Bai Y., Yao B. (2019). Synergistic Effect of Acetyl Xylan Esterase from Talaromyces Leycettanus JCM12802 and Xylanase from Neocallimastix Patriciarum Achieved by Introducing Carbohydrate-Binding Module-1. AMB Express.

[28] Kato T., Shiono Y., Koseki T. (2021). Identification and Characterization of an Acetyl Xylan Esterase from Aspergillus Oryzae. J. Biosci. Bioeng..

[29] Zhang Y., Ding H.-T., Jiang W.-X., Zhang X., Cao H.-Y., Wang J.-P., Li C.-Y., Huang F., Zhang X.-Y., Chen X.-L. (2021). Active Site Architecture of an Acetyl Xylan Esterase Indicates a Novel Cold Adaptation Strategy. J. Biol. Chem..

[30] Sista Kameshwar A.K., Qin W. (2018). Understanding the Structural and Functional Properties of Carbohydrate Esterases with a Special Focus on Hemicellulose Deacetylating Acetyl Xylan Esterases. Mycology.

[31] Burlacu A., Israel-Roming F., Cornea C.P. (2018). Screening of microorganisms displaying acetyl xylan esterase activity. Sci. Pap. Ser. B Hortic..

[32] Rashamuse K., Ronneburg T., Sanyika W., Mathiba K., Mmutlane E., Brady D. (2014). Metagenomic Mining of Feruloyl Esterases from Termite Enteric Flora. Appl. Microbiol. Biotechnol..

[33] Ferrer M., Golyshina O.V., Chernikova T.N., Khachane A.N., Reyes-Duarte D., Martins Dos Santos V.A.P., Strompl C., Elborough K., Jarvis G., Neef A. (2005). Novel Hydrolase Diversity Retrieved from a Metagenome Library of Bovine Rumen Microflora. Environ. Microbiol..

[34] Adesioye F.A., Makhalanyane T.P., Biely P., Cowan D.A. (2016). Phylogeny, Classification and Metagenomic Bioprospecting of Microbial Acetyl Xylan Esterases. Enzym. Microb. Technol..

[35] Razeq F.M., Jurak E., Stogios P.J., Yan R., Tenkanen M., Kabel M.A., Wang W., Master E.R. (2018). A Novel Acetyl Xylan Esterase Enabling Complete Deacetylation of Substituted Xylans. Biotechnol. Biofuels.

[36] Peláez M.L., Valdecasas A.G., Martinez D., Horreo J.L. (2018). Towards the Unravelling of the Slug A. Ater-A. Rufus Complex (Gastropoda Arionidae): New Genetic Approaches. Web Ecol..

[37] Joynson R., Pritchard L., Osemwekha E., Ferry N. (2017). Metagenomic Analysis of the Gut Microbiome of the Common Black Slug Arion Ater in Search of Novel Lignocellulose Degrading Enzymes. Front. Microbiol..

[38] Wang Z., Pawar P.M.A., Derba-Maceluch M., Hedenström M., Chong S.L., Tenkanen M., Jönsson L.J., Mellerowicz E.J. (2020). Hybrid Aspen Expressing a Carbohydrate Esterase Family 5 Acetyl Xylan Esterase Under Control of a Wood-Specific Promoter Shows Improved Saccharification. Front. Plant Sci..

[39] Yang Y., Yang J., Liu J., Wang R., Liu L., Wang F., Yuan H. (2018). The Composition of Accessory Enzymes of Penicillium Chrysogenum P33 Revealed by Secretome and Synergistic Effects with Commercial Cellulase on Lignocellulose Hydrolysis. Bioresour. Technol..

[40] Kim M.J., Jang M.U., Nam G.H., Shin H., Song J.R., Kim T.J. (2020). Functional Expression and Characterization of Acetyl Xylan Esterases CE Family 7 from Lactobacillus Antri and Bacillus Halodurans. J. Microbiol. Biotechnol..

[41] McKay A.M. (1993). Microbial Carboxylic Ester Hydrolases (EC 3.1.1) in Food Biotechnology. Lett. Appl. Microbiol..

[42] Bornscheuer U.T. (2002). Microbial Carboxyl Esterases: Classification, Properties and Application in Biocatalysis. FEMS Microbiol. Rev..

[43] Kyu Kim K., Kyu Song H., Hae Shin D., Yeon Hwang K., Choe S., Joon Yoo O., Won Suh S. (1997). Crystal Structure of Carboxylesterase from Pseudomonas Fluorescens, an a/b Hydrolase with Broad Substrate Specificity. Structure.

[44] Khan F.I., Lan D., Durrani R., Huan W., Zhao Z., Wang Y. (2017). The Lid Domain in Lipases: Structural and Functional Determinant of Enzymatic Properties. Front. Bioeng. Biotechnol..

[45] Verger R. (1997). ‘Interfacial Activation’ of Lipases: Facts and Artifacts. Trends Biotechnol..

[46] Sood S., Sharma A., Sharma N., Kanwar S.S. (2018). Carboxylesterases: Sources, Characterization and Broader Applications. Insights Enzym. Res..

[47] Li X., Griffin K., Langeveld S., Frommhagen M., Underlin E.N., Kabel M.A., de Vries R.P., Dilokpimol A. (2020). Functional Validation of Two Fungal Subfamilies in Carbohydrate Esterase Family 1 by Biochemical Characterization of Esterases From Uncharacterized Branches. Front. Bioeng. Biotechnol..

[48] Ali S., Mahmood S. (2020). Mutagenesis of a Thermophilic Alkalibacillus Flavidus for Enhanced Production of an Extracellular Acetyl Xylan Esterase in Semi-Solid Culture of Linseed Meal. Waste Biomass Valorization.

[49] Qaseem M.F., Wu A.M. (2020). Balanced Xylan Acetylation Is the Key Regulator of Plant Growth and Development, and Cell Wall Structure and for Industrial Utilization. Int. J. Mol. Sci..

[50] Dilokpimol A., Verkerk B., Bellemare A., Lavallee M., Frommhagen M., Underlin E.N., Kabel M.A., Powlowski J., Tsang A., de Vries R.P. (2020). Characterization of New Fungal Carbohydrate Esterase Family 1 Proteins Leads to the Discovery of Two Novel Dual Feruloyl/Acetyl Xylan Esterases. https://www.researchsquare.com/article/rs-17222/latest.pdf.

[51] Yin C.-F., Xu Y., Deng S.-K., Yue W.-L., Zhou N.-Y. (2021). A Novel Esterase, DacA Pva, from *Comamonas* Sp. Strain NyZ500 with Deacetylation Activity for the Acetylated Polymer Polyvinyl Alcohol. Appl Environ Microbiol..

[52] Urbanek A.K., Mirończuk A.M., García-Martín A., Saborido A., de la Mata I., Arroyo M. (2020). Biochemical Properties and Biotechnological Applications of Microbial Enzymes Involved in the Degradation of Polyester-Type Plastics. Biochim. Et Biophys. Acta-Proteins Proteom..

[53] Koseki T., Furuse S., Iwano K., Sakai H., Matsuzawa H. (1997). An Aspergillus Awamori Acetylesterase: Purification of the Enzyme, and Cloning and Sequencing of the Gene. Biochem. J..

[54] Chung H.-J., Park S.-M., Kim H.-R., Yang M.-S., Kim D.-H. (2002). Cloning the Gene Encoding Acetyl Xylan Esterase from Aspergillus Ficuum and Its Expression in Pichia Pastoris. Enzym. Microb. Technol..

[55] Koseki T., Miwa Y., Akao T., Akita O., Hashizume K. (2006). An Aspergillus Oryzae Acetyl Xylan Esterase: Molecular Cloning and Characteristics of Recombinant Enzyme Expressed in Pichia Pastoris. J. Biotechnol..

[56] Degrassi G., Okeke B.C., Bruschi C.V., Venturi V. (1998). Purification and Characterization of an Acetyl Xylan Esterase from Bacillus Pumilus. Appl. Environ. Microbiol..

[57] Levisson M., Han G.W., Deller M.C., Xu Q., Biely P., Hendriks S., ten Eyck L.F., Flensburg C., Roversi P., Miller M.D. (2012). Functional and Structural Characterization of a Thermostable Acetyl Esterase from Thermotoga Maritima. Proteins: Struct. Funct. Bioinform..

[58] Drzewiecki K., Angelov A., Ballschmiter M., Tiefenbach K.J., Sterner R., Liebl W. (2010). Hyperthermostable Acetyl Xylan Esterase. Microb. Biotechnol..

[59] Shao W., Wiegel J. (1995). Purification and Characterization of Two Thermostable Acetyl Xylan Esterases from *Thermoanaerobacterium* sp.. Strain JW/SL-YS485. Appl. Environ. Microbiol..

[60] Kumar S., Stecher G., Li M., Knyaz C., Tamura K. (2018). MEGA X: Molecular Evolutionary Genetics Analysis across Computing Platforms. Mol. Biol. Evol..

[61] Ontañon O.M., Ghio S., Marrero Díaz de Villegas R., Piccinni F.E., Talia P.M., Cerutti M.L., Campos E. (2018). EcXyl43 β-Xylosidase: Molecular Modeling, Activity on Natural and Artificial Substrates, and Synergism with Endoxylanases for Lignocellulose Deconstruction. Appl. Microbiol. Biotechnol..

[62] Yang J., Zhang Y. (2015). Protein Structure and Function Prediction Using I-TASSER. Curr. Protoc. Bioinform..

[63] Wierzbicka-Woś A., Henneberger R., Batista-García R.A., Martínez-Ávila L., Jackson S.A., Kennedy J., Dobson A.D.W. (2019). Biochemical Characterization of a Novel Monospecific Endo-β-1,4-Glucanase Belonging to GH Family 5 from a Rhizosphere Metagenomic Library. Front. Microbiol..

