# Supplementary material for: Characterisation of a Novel Acetyl Xylan Esterase (BaAXE) Screened from the Gut Microbiota of the Common Black Slug (Arion ater)
Source: Molecules. 2022 May 7;27(9):2999. doi: 10.3390/molecules27092999 (PMC9104356; doi:10.3390/molecules27092999)
Supplement: Supplementary file 1 [file molecules-27-02999-s001.zip › molecules-1683451-supplementary.pdf]

Supplementary Data.

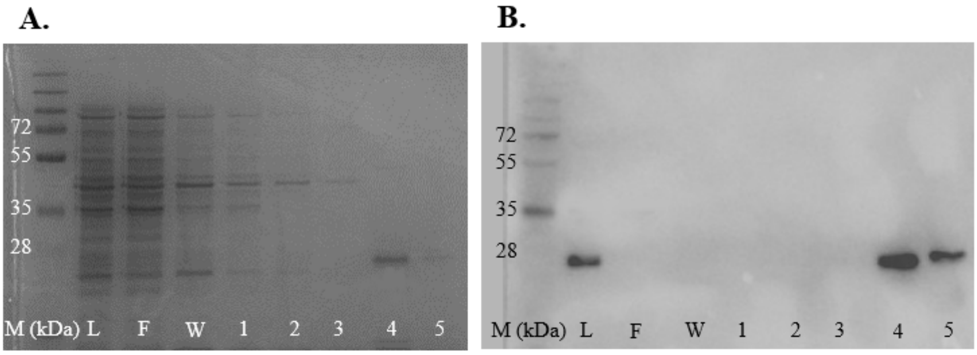

Figure S1. Purification of Gene_id_40363. Proteins from lysed cells were passed through HisTalon gravity columns and eluted with stepwise concentrations of imidazole. Aliquots of the lysate (L), flow-through (F), wash (W) and elutions (lanes 1-5) were analysed on SDS-PAGE (**A**) and western blot (**B**). Elution fractions 1-5 represents 20%, 40%, 60%,80 % and 90% of a 150 mM imidazole elution buffer (50 mM sodium phosphate, 150 mM NaCl) respectively.

**Method file**	D:\MassHunter\GCMS\1\methods\Henry 50-290, 15min, 150C inlet 10 spilt.M
**Tune file**	ATUNE.U
**Ion source**	EI
**Source temperature (°C)**	230
**Quad temperature (°C)**	150
**Fixed Electron energy (eV)**	70.0
**Acquisition Type**	Scan
**Stop time (min)**	10.00
**Solvent delay (min)**	2.00
**Trace Ion Detection**	False
**Gain Factor**	1
**EM Saver**	False
**EM Saver Limit**	N/A

Scan Time Segments

Time	Start Mass	End Mass	Threshold	Scan Speed
2.00	40	200	150	1,562 [N=2]

Figure S2. MS Parameter Report.
